# Community Science Strategies Reveal Distributional Patterns of Treponeme-Associated Hoof Disease in Washington Elk (*Cervus canadensis*)

**DOI:** 10.1155/2023/6685108

**Published:** 2023-12-14

**Authors:** Steven N. Winter, María del Pilar Fernández, Erin Clancey, Kyle Garrison, Kristin Mansfield, Margaret A. Wild

**Affiliations:** ^1^Department of Veterinary Microbiology and Pathology, Washington State University, P.O. Box 647040, Pullman, WA 99164, USA; ^2^Paul G. Allen School for Global Health, Washington State University, P.O. Box 647090, Pullman, WA 99164, USA; ^3^Washington Department of Fish and Wildlife, P.O. Box 43141, Olympia, WA 98504, USA

## Abstract

Treponeme-associated hoof disease (TAHD) is an emerging disease of conservation concern in elk (*Cervus canadensis*) in the Northwest USA. Elk with TAHD exhibit characteristic hoof lesions that are often accompanied by lameness and limping. The gold standard approach traditionally used for infectious disease surveillance is laboratory confirmation, which for TAHD is a histologic examination of abnormal elk hooves submitted by wildlife agencies. Diagnostic evaluation affords certainty in confirming TAHD; however, these examinations are also labor and resource intensive, and therefore, not conducive to the collection of sufficient data for epidemiologic investigations. In response, two community science (CS) surveillance strategies have been implemented in Washington State: public observations of limping elk from a web-based reporting tool and hunter reports of hoof abnormalities on harvested elk. Surveillance using CS strategies can be implemented widely and may be useful for describing broad distributional patterns of TAHD, despite their unknown relationship to laboratory-confirmed cases. We described and compared the spatial–temporal distribution of TAHD in western Washington game management units (GMU) using the two CS strategies to assess congruences and discrepancies between observed patterns. We used spatial scan statistics to identify possible core-affected and newly emerging areas at the GMU level. Lastly, we contrasted CS observations against confirmed case data to examine possible delays in TAHD detection and co-occurrence among surveillance strategies. We found public observations of limping elk often predated TAHD confirmations in GMUs by several years, while hunter-reported abnormalities predated confirmations in GMUs by several months. High co-occurrence between the presence of apparent and confirmed cases under different surveillance strategies further supports the use of CS sources. This study capitalizes on wide-reaching CS data to provide new and complementary epidemiological information that can help guide future surveillance, management, and research efforts for this novel elk hoof disease.

## 1. Introduction

Emerging diseases in wildlife are challenging to monitor and manage, in part because wildlife populations are often spread over vast or remote areas and are difficult to observe and sample. As a result, data on the abundance and distribution of affected wildlife populations are often limited, making it difficult to track the spread of disease and implement control measures [1]. Timely management responses are also often expected or required despite limited disease surveillance data or scientific knowledge about the biological and ecological factors associated with disease transmission. Ideally, researchers have access to large surveillance datasets, which are key to assess the distribution of disease in space and time and understand disease transmission dynamics with diverse analytical techniques [2]. For example, such datasets have been used to identify high-risk areas where disease occurrence might be aggregated [3] or determine demographic and environmental drivers of transmission [1, 4, 5]. However, acquiring large datasets for such analyses can be labor and resource intensive, particularly when surveillance approaches are reliant on laboratory diagnosis of disease. Thus, identifying cost-effective surveillance methods that can be readily applied to emerging wildlife diseases is critical to support evidence-based management.

Community science (CS) strategies can serve as low-cost, wide-reaching data collection methods for wildlife disease surveillance and monitoring [6, 7]. Community (or citizen) science broadly describes the concept of conducting scientific research using data collected by volunteers who may not necessarily be trained scientists. Community science is common in ecology because many wildlife species and ecological processes can be sampled or observed by both scientists and nonscientists, such as hunters, landowners, and other local observers [6, 8, 9]. For instance, even simple public observations of wildlife may be effective for surveilling and improving understanding of some diseases, particularly those characterized by obvious gross deformities or disabilities [7, 10, 11]. Community science strategies offer complementary information to data collected directly by wildlife managers [12] and increase the geographic extent of surveyable areas, including public and private lands. Nevertheless, caution is warranted when interpreting results from CS strategies [8], particularly for hypothesis-driven research [13]. Sampling biases from uneven participant interest and training over time or concentration of records near human settlements or roadways [14] may affect the accuracy of inferences [15]. Similarly, diseased individuals may become more vulnerable to sampling from hunter harvest [16] or opportunistic mortalities [17, 18]. Thus, researchers often use expert-collected data (when available) to identify and adjust for biases in CS data [19–21].

In 2008, the Washington Department of Fish and Wildlife (WDFW) received an increase in public reports of limping elk (*Cervus canadensis*) and elk with hoof abnormalities in southwestern Washington, USA, although some reports date back to the early 2000s. Field investigations in this index area revealed a local abundance of elk with unique hoof lesions, including severe sole ulcerations and overgrown, deformed, and sloughed hoof capsules, of unknown etiology [22, 23]. Further investigations identified an association with *Treponema* spp. bacteria in lesions [24], leading to the designation as treponeme-associated hoof disease (TAHD; [25]). Recent experimental challenges provided direct evidence that TAHD is an infectious, transmissible disease in elk [26] and that lesions follow a predictable progression of gross deformities [25, 26] directly related to lameness and limping in captive elk [26]. The gold standard strategy traditionally used for surveillance of infectious diseases in wildlife involves diagnostic confirmation of pathogens in samples that are usually submitted by wildlife management agencies. In the case of TAHD, confirmation is conducted using histologic examination to identify characteristic suppurative inflammation and the presence of spirochetes using silver stain [25]. Enhanced surveillance efforts using this strategy led to the confirmation of TAHD in four Pacific Northwest states: Washington, Idaho, Oregon, and California [27]. Although such surveillance data (hereafter, “Confirmed Cases”) provide evidence-based information and can be used to describe the geographic range of TAHD in a broad sense, they are not conducive to identifying spatial–temporal patterns for improving TAHD management due to the current resource-intensive diagnostic approach. Because elk with TAHD exhibit hoof abnormalities and associated limping, WDFW developed two CS surveillance strategies to capture additional TAHD “cases” from: (i) public observations of limping elk from a web-based reporting tool and (ii) hunter reports of hoof abnormalities in harvested elk.

Surveillance data collected under WDFW's CS strategies lack laboratory-confirmed diagnoses; however, they may still be useful for exploring general distributional patterns of TAHD and guiding hypothesis formation [13]. In this study, we aimed to describe broad spatial and temporal distributions of TAHD based on CS data in Washington. We investigated congruences and discrepancies in observed patterns in TAHD surveillance data across western Washington game management units (GMUs) over time, including a 6-year span when CS collection periods overlapped (2016–2021). We used the spatial and temporal patterns derived from the CS data to identify apparent core-affected and newly emerging areas of TAHD, as well as time periods with higher occurrence of apparent cases. Lastly, we contrasted CS data with Confirmed Case data to examine possible delays in confirmed TAHD detection and co-occurrence between different surveillance strategies. In doing so, we identify parallel and complementary epidemiological value in existing datasets and provide useful information and insights for guiding future TAHD surveillance, management, and research.

## 2. Materials and Methods

### 2.1. TAHD Surveillance Data Sources

A web-based reporting tool was one of the two CS surveillance data sources evaluated. The web-based tool allows the public to record and geolocate observations of limping elk and dead elk with hoof abnormalities (Figure S1; [28]). The WDFW-maintained website has been continuously accessible upon web search since its inception in late 2012 (Table 1). From 2012 to 2017, WDFW promoted the website at public meetings and in hunting regulation pamphlets. More recently (2017–present), WDFW advertised the website in various outreach materials (e.g., pamphlets and presentations) and by word of mouth. Available data on how reporting parties heard about the tool indicated most were directed from the WDFW website (60%), followed by WDFW representatives (15%), social media (5%), news outlets (1%), and other unspecified routes (19%) (WDFW, *Unpubl. data*). For the current study, we removed records describing dead elk with hoof abnormalities (8.5% of total) to retain only observations of limping elk; we refer to these data as “Public Observations.” Although Public Observations contained coordinates for estimated counts of total elk observed and the number seen limping, we converted these cases to simple presence-only records and upscaled them to the GMU-level to minimize opportunities for errors in estimated counts, geolocations, or observer effects [29].

The second CS surveillance strategy relied upon reports from hunters and was previously described by Wild et al. [30]. Briefly, WDFW collected data using mandatory hunter reports to assess whether harvested elk had hoof abnormalities. These data, referred to as “Hunter Reports” (Table 1), are collected statewide at the GMU-level and contain individual elk information, including harvest details and disease case data in the form of hunters' responses of “Yes,” “No,” or “Unknown” to the question: “In your opinion, did any of the hooves from the elk you harvested appear to be deformed or exhibit any abnormalities?” The WDFW educated hunters on TAHD using written and illustrated materials before harvest seasons through hunting regulations and email less than 1 week before the first day of elk season. Hunter Reports for harvest years 2018–2021 were collected near the end of the year's harvest. Data from 2016 and 2017 harvest seasons were retrospectively collected in 2018 and relied on hunter recall of the previous 2 years. Omitting “Unknown” responses (0.8% of total), we refer to the proportion of cases (i.e., hoof abnormalities) in the total number of reports in a given GMU as the “apparent” TAHD prevalence of the GMU because samples did not undergo diagnostic testing.

Finally, “Confirmed Case” data were obtained from field collection of hooves that were examined histologically to diagnose TAHD (Table 1). These data were discussed in length by Wild et al. [27]. Briefly, abnormal hooves were opportunistically submitted by hunters to wildlife management agencies or collected from elk that were culled for research or management actions since approximately 2009 [22, 27]. Hooves were submitted to veterinary diagnostic laboratories for case confirmation by a veterinary pathologist. Confirmed Cases from 2009 to 2014 were largely collected for diagnostic evaluation and disease description purposes [22, 24, 25]. Subsequently, Confirmed Cases through 2021 were solicited to expand sampling coverage for delineating the extent of TAHD's regional distribution [27]. Due to the sampling approach, these data are not representative of TAHD prevalence [27]; they most appropriately serve the current study as evidence of TAHD being confirmed in certain GMUs.

### 2.2. Study Area

We delineated our study area as all GMUs that contained documented elk herd areas in western Washington, i.e., “areas west of the Pacific Crest Trail and west of (and including) the Big White Salmon River in Klickitat and Skamania counties” [32]. These portions of the state correspond to areas where TAHD was first detected and contain higher concentrations of surveillance data ([27]; Figure S2). Data analyses were focused on this study area.

### 2.3. Spatial–Temporal Patterns of CS Data

#### 2.3.1. Distributions and Trends of Public Observations

We mapped Public Observations over time at the GMU-level to document the spatial coverage of limping elk reports across the study area. Public Observation data were reported throughout the year and included the date of the report (Table 1). This allowed us to investigate the seasonality of observations by evaluating the monthly count of limping elk observations within the study area via evidence of temporal autocorrelation using the acf function in the R stats package ([33]; v4.0.5). We also assessed whether the frequency of Public Observations across the study area broadly remained consistent over time using univariate linear regression, where the number of monthly observations was the response variable and time was the predictor variable.

#### 2.3.2. Distributions and Trends of Hunter-Reported Prevalence

We calculated and mapped the crude apparent prevalence from Hunter Reports (hereafter, Hunter-Reported prevalence) on an annual basis at the GMU-level to explore baseline prevalence and trends across units. However, given the high variability in sample sizes among GMUs, we further evaluated general trends in herd-level prevalence over time by aggregating Hunter Reports from GMUs into their larger corresponding elk herd areas ([32]; Figure S2). We calculated crude annual herd-level prevalence and modeled prevalence over time with a binomial generalized linear model (GLM). We held prevalence as the dependent variable and included an interactive effect between herd and harvest year as covariates to test for differences in prevalence in herds over time. Lastly, given management interests, we also evaluated differences in apparent prevalence between sexes, using a chi-square test and a binomial GLM to calculate odds ratios (OR) with 95% confidence intervals (CIs).

#### 2.3.3. Spatial Clusters in CS Strategies

We described the spatial distribution of CS data within the study area by first evaluating the extent of spatial aggregation of cases from each CS strategy by calculating the Moran's *I* statistic with the R package spdep [34], which assesses the spatial autocorrelation in the number of events. We also plotted distances at which spatial autocorrelation occurred with univariate correlograms in the R package ncf [35].

Next, we examined whether signs consistent with TAHD occurred in local spatial or spatial–temporal clusters using spatial scan statistics [36–38]. We used Kulldorff spatial scan statistics in SaTScan software (v10.2, http://www.satscan.org), which evaluated the number of apparent cases counted within multiple circular (or cylindrical) windows. Using an inferred spatial process that results in an expected number of cases, we compared the observed number of cases against those outside of the scanning window using likelihood ratio testing. The scanning window that maximized the log-likelihood ratio was defined as the most likely (i.e., primary) local cluster [39, 40]. We tailored the type of analysis and spatial process probability model for identifying statistically significant clusters to the type of CS data described above. More specifically, we tested for evidence of local clusters in the number of hoof abnormalities given the underlying harvested elk population in Hunter Reports with the Poisson purely spatial scan statistic [39]. By contrast, we used the retrospective space-time permutation model to assess spatial–temporal clusters in monthly Public Observations at the GMU-level because we did not have data for the underlying elk population abundance from which Public Observations were made [40]. For parameterizing all models and calculating statistical significance, we limited maximum cluster size to 50% of the population and used 999 replicates of Monte Carlo simulations to generate cluster-specific *p*-values; for space-time tests, we limited temporal clusters to no more than 50% of the study duration. Ultimately, we defined GMUs that contained primary clusters under both CS strategies as potential “core-affected” GMUs. We also report statistically significant secondary clusters that did not overlap with primary clusters to identify other areas of concern, such as locations where TAHD may be newly emerging [39].

#### 2.3.4. Comparison of the Number of CS Cases

Washington's CS strategies overlapped during the 2016–2021 harvest seasons, allowing us to compare the number of apparent cases in GMUs reported in each of these datasets. However, given the differences in spatial and temporal resolutions of CS datasets (Table 1), we grouped Public Observation data to an annual harvest season basis at the GMU-level so they could be compatible with the format of data from Hunter Reports. That is, observations of limping elk that occurred after the conclusion of a harvest season were grouped with the subsequent season.

We evaluated the extent to which the number of apparent cases from Hunter Reports and Public Observations correlated and agreed with each other on an annual basis at the GMU level. We calculated Spearman's rank correlations (*r*) to quantify the correlation between the number of cases as well as the relationship between Hunter-Reported prevalence and the number of Public Observations. To evaluate agreements (i.e., the similarity between measurements) between the number of apparent cases, we constructed Bland–Altman plots (Tukey mean difference plots), which have been used for comparing observations in CS programs [9]. We calculated both the means of $\left(\mu_{i,j}=\frac{\left({HR}_{i,j}+{PO}_{i,j}\right)}{2}\right)$ and differences between (*d*_*i*,*j*_=HR_*i*,*j*_ − PO_*i*,*j*_) the number of cases from CS strategies in GMUs over time, where HR and PO represent the number of apparent cases from Hunter Reports and Public Observations, respectively, in a given GMU, *i*, for harvest year, *j*. After verifying differences were normally distributed with a Shapiro–Wilk test [41], we calculated the mean difference ($\overset{―}{d}$) and standard deviation of differences (*s*) to define the limits of agreement (LoA; $\overset{―}{d}\pm 2\times s$). Following Bland and Altman [42], we considered CS strategies to be in agreement with each other if ≥95% of their differences were within the LoA.

### 2.4. Contrasting Confirmed Case Data against CS Patterns

We aimed to identify possible delays in confirmed TAHD detection when surveillance strategies overlapped in time by contrasting the harvest year in which CS strategies first detected apparent cases to the harvest year in which the first Confirmed Case was described in each GMU. For this, we calculated the average difference between the earliest years of CS detections from the earliest year of a Confirmed Case in GMUs. Additionally, to examine the general co-occurrence of detections under the different surveillance strategies, we coded the occurrence of apparent cases from the two CS strategies and the occurrence of Confirmed Cases as simple presence–absence data at the GMU-level. These data were placed in contingency tables for Fisher's exact tests. Next, we qualitatively compared the GMUs that comprised the core-affected area as well as primary and secondary clusters from Section 2.3.3 and the geographic distribution of Confirmed Case data. Finally, because Bland–Altman plots can indicate GMUs that were outliers of agreement (calculated in Section 2.3.4), we evaluated the confirmation status of GMUs that occurred outside of the LoA to explore if a history of confirmed TAHD corresponded with agreement between CS strategies.

## 3. Results

### 3.1. Distributions and Trends of Public Observations

**Figure 1:**
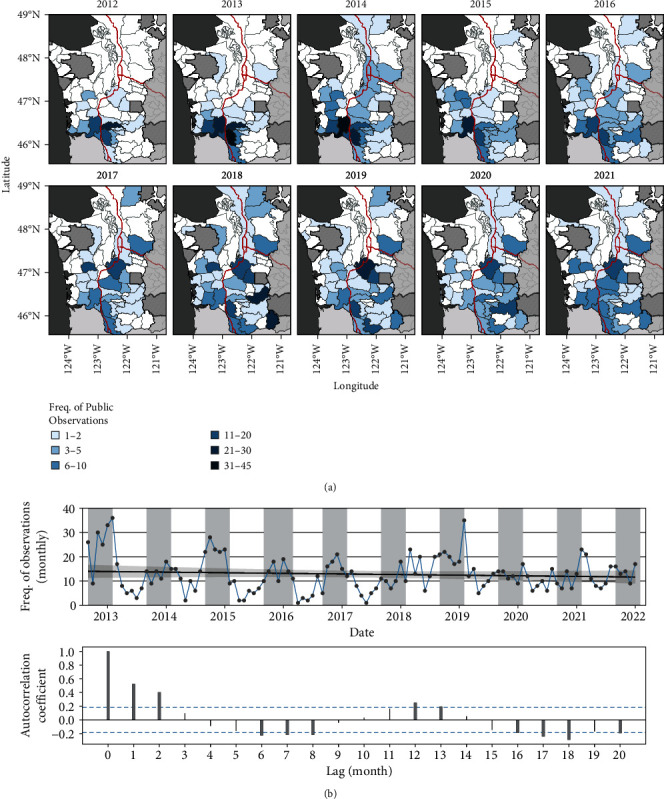
Expanding spatial distribution and seasonality of Public Observations of limping elk. (a) Maps show the expanding spatial distribution of Public Observations from 2012 to 2021. Darker blue colors in polygons represent relatively larger numbers of observations at an annual scale. Areas outside the Washington Department of Fish and Wildlife jurisdiction are shown in striped polygons; neighboring states (Oregon to south) and other GMUs outside of the study area in gray. Interstate highways shown in thick red lines are included as landmarks. (b) Time series displays signs of a cyclic, seasonal pattern in the monthly number of Public Observations from late 2012 to early 2022 (i.e., the 2021 harvest season). Gray portions of the time series represent harvest season months (September–January). The pattern of annual peaks aligned with thicker bars in the autocorrelation plot (bottom panel), indicating statistically significant monthly lags that would be consistent with seasonality in reporting. Public Observations are displayed as raw, unadjusted values because the observer population, sampling effort, and detection probabilities were unknown.

The spatial distribution of Public Observations expanded from southwestern Washington to most GMUs in the study area during the 2012–2021 harvest years (Figure 1(a)). Temporal autocorrelation analyses revealed temporal dependence in the monthly frequency of Public Observations at 6-month intervals (i.e., 6, 12, 18 months). Reports increased prior to and peaked during harvest months (i.e., September–January) (Figure 1(b)). The univariate linear regression model revealed a decreasing but not significant trend (*p*-value > 0.05) in the number of monthly observations over time during the study period.

### 3.2. Distributions and Trends of Hunter-Reported Prevalence

**Figure 2:**
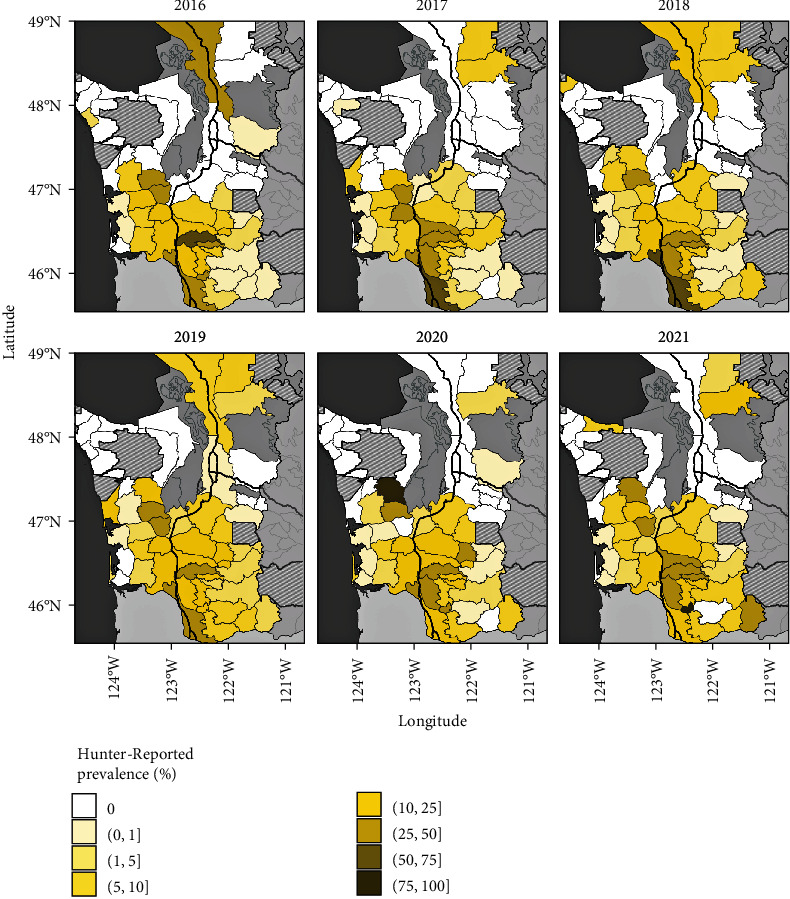
Spatial–temporal distribution of Hunter-Reported prevalence. Western Washington game management unit (GMU) maps show raw estimates of the apparent prevalence of reports of hoof abnormalities collected annually by hunters during the 2016–2021 harvest seasons. Proportions were converted to percentages and binned for visualizations. Prevalence estimates are not adjusted by sample sizes and thus do not reflect uncertainty in estimates. Areas outside the Washington Department of Fish and Wildlife jurisdiction are shown in striped polygons; neighboring states (Oregon to south) and other GMUs outside of the study area in gray. Interstate highways shown in black lines are included as landmarks.

**Figure 3:**
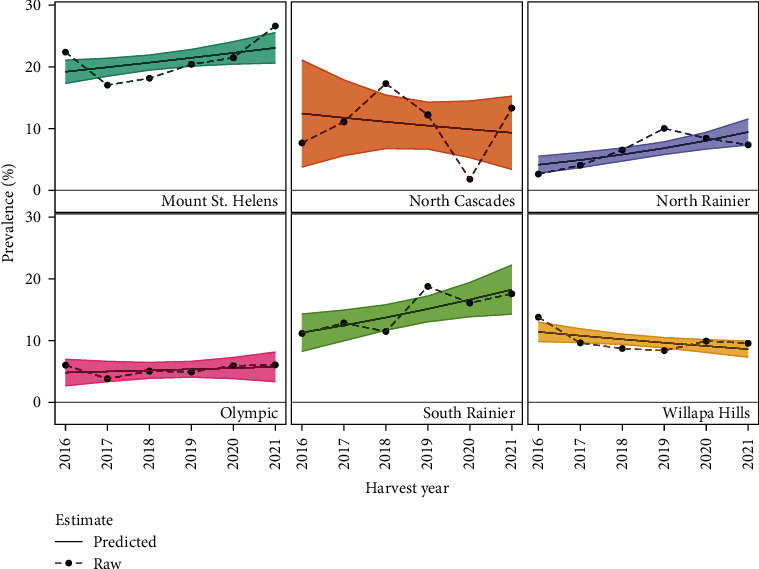
Varying trends in Hunter-Reported prevalence over time in western Washington elk herd areas. We evaluated trends in Hunter-Reported prevalence aggregated to game management units comprising designated elk herd areas (panels). Raw annual prevalence estimates are connected by dotted lines. Colored solid lines with ribbons show slopes and confidence intervals from a binomial generalized linear model. We identified significant positive slopes in prevalence over time for Mount St. Helens and North Rainier elk herd areas. We found a significant decreasing trend in prevalence in the Willapa Hills elk herd area. Effects of time on prevalence were less clear for Olympic, North Cascades, and South Rainier herd areas.

Hunter Reports of hoof abnormalities occurred in most (80%) GMUs of the study area (Figure 2). Of the GMUs with apparent cases, the median crude annual prevalence was 11.58%. When analyzed by herd area, we detected several significant trends in prevalence over time (Figure 3). We observed a non-zero positive slope in prevalence in North Rainier and Mount St. Helens herd areas and a non-zero negative slope in prevalence in elk from the Willapa Hills herd area (Figure 3). Trends in prevalence over time for the Olympic, South Rainier, and North Cascades herd areas were not statistically clear (Table S1). Finally, we identified a significant difference in apparent prevalence between sexes (*χ*^2^ = 7.984, *df* = 1, *p*-value = 0.005), with female elk having slightly higher relative odds for hoof abnormalities (OR = 1.16, 95% CI: 1.048–1.290).

### 3.3. Spatial Clusters in CS Strategies

We found significant positive spatial autocorrelation in the number of apparent cases from CS strategies at the GMU-level (Public Observations Moran's *I*: 0.309, *p*-value < 0.001; Hunter Reports Moran's *I*: 0.572, *p*-value < 0.001). Similarly, correlograms revealed consistent positive spatial autocorrelation at smaller lagged distances for Hunter-Reported hoof abnormalities; by comparison, significant spatial autocorrelation in Public Observations was widespread across the study area (Figure S3).

**Figure 4:**
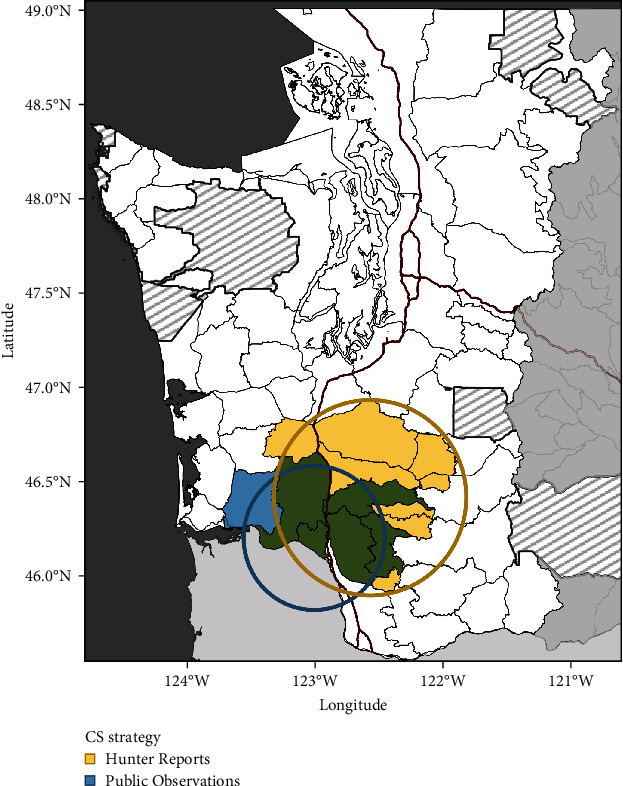
Core-affected game management units based on community science data. Blue polygons represent game management units (GMUs) that contained the most likely space-time cluster from Public Observations (blue circle). Yellow polygons represent the GMUs contained within the most likely spatial cluster from Hunter Reports (yellow circle). Defined as core-affected GMUs, five GMUs (green polygons) show the spatial overlap between both primary clusters on either side of the interstate highway (red lines). Striped polygons represent jurisdictions outside of Washington Department of Fish and Wildlife GMUs, including tribal reservations and national parks.

We identified primary clusters from each CS dataset (i.e., those with the highest log-likelihood ratios) consisting of 14 unique GMUs in southwestern Washington (Figure 4). The primary cluster of Hunter Reports contained GMUs in elk herd areas of Mount St. Helens, Willapa Hills, and South Rainier (Figure S2). The primary space-time cluster of Public Observations consisted of six GMUs in elk herd areas of Willapa Hills and Mount St. Helens (Figure 4). As we defined the core-affected area as the GMUs where CS primary clusters overlap, five contiguous GMUs in southwestern Washington were considered the core-affected area (Figure 4). We also detected multiple secondary clusters in CS data distant from the south-central portion of the study area (Figure S4).

### 3.4. Comparison of the Number of CS Cases

**Figure 5:**
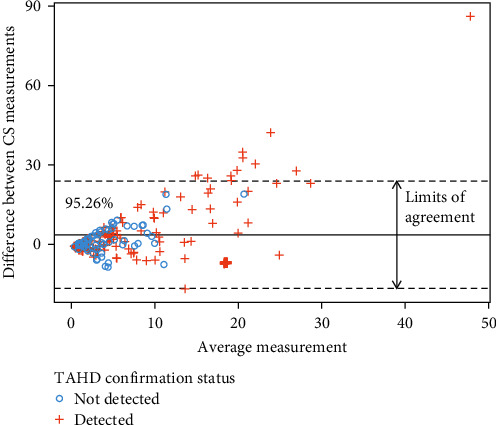
Agreements between the annual number of cases from community science (CS) strategies. Bland–Altman plot shows differences between the number of apparent cases measured from CS strategies (*y* axis) and the average measurement of the number of apparent cases on an annual basis at the GMU level (*x* axis). Greater than 95% (95.26%) of differences between measurements were between limits of agreement (dashed lines), which is indicative of good agreement between surveillance data sources.

We found a moderate, significant correlation between the number of Public Observations and the number of Hunter Reports on an annual basis at the GMU-level (Spearman *r* = 0.513, *p*-value < 0.001), but a weaker relationship between crude annual Hunter-Reported prevalence and number of Public Observations (Spearman *r* = 0.301, *p*-value < 0.001). Further, results from Bland–Altman analysis revealed 95.27% of differences in the number of apparent CS cases were within the LoA (Figure 5).

### 3.5. Contrasting Confirmed Case Data against CS Patterns

**Figure 6:**
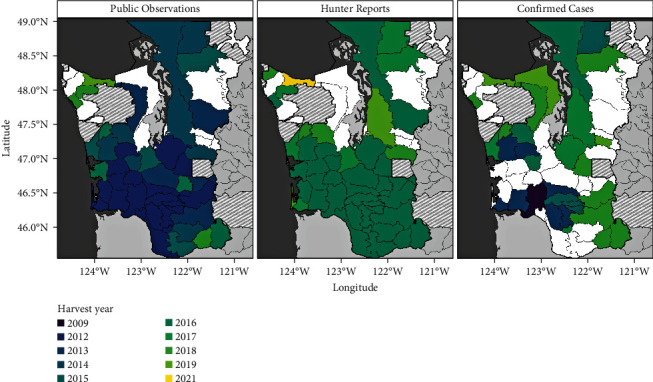
Maps of harvest years with earliest game management unit-level detection from TAHD surveillance strategies. Panels show the presence of apparent cases or Confirmed Cases collected under different TAHD surveillance strategies (panel labels) in western Washington game management units (GMUs). GMUs were colored to represent older (dark blue) to more recent (yellow) harvest years in which respective cases were first identified. For community science strategies, many detections were first recorded in the year they were developed (i.e., Public Observations—2012, Hunter Reports—2016).

We found the earliest year of Public Observations often predated Confirmed Cases in a GMU, with a mean difference of 2.32 years. Similarly, Hunter Reports often predated Confirmed Cases with a mean difference of 0.56 years (Figure 6). While we identified a significant association between the presence of Confirmed Cases and Public Observations at the GMU level (*p*-value = 0.008, Fisher's exact test), the presence of apparent cases from Hunter Reports and Confirmed Cases were not significantly associated (*p*-value = 0.094, Fisher's exact test). We also identified significant co-occurrence between both CS strategies (*p*-value < 0.001, Fisher's exact test) at the GMU-level. We observed the highest concurrence of Confirmed Cases and apparent cases derived from CS data in primary clusters and core-affected GMUs identified in the previous spatial analyses (Figure 4). Confirmed Cases were present in 5/6 (83.3%) and 8/13 (69.2%) GMUs comprising the primary clusters based on Public Observations and Hunter Reports, respectively, and Confirmed Cases were present in 4/5 (80%) of core-affected GMUs (defined as the areas in which both primary clusters overlap). By contrast, lower concurrence was found in secondary clusters (which are potential areas of newly emerging disease occurrence): Confirmed Cases were described in 12/21 (57.1%) of the GMUs included in the secondary clusters identified using Public Observations, and in 9/15 GMUs (60%) of the secondary clusters identified using Hunter Reports (Figure S4). Finally, all GMUs that were outside of the LoA in the Bland–Altman plot comparing the agreement between the number of apparent cases in CS strategies had confirmed TAHD (Figure 5).

## 4. Discussion

Community science strategies revealed distributional patterns and trends in elk with signs of TAHD within the study area that were not evident using Confirmed Cases alone and that are worthy of exploration in future studies. We highlight three main findings from our analyses: (i) apparent cases occurred in spatial clusters and expanded from southwestern Washington to most GMUs in the study area, (ii) the two CS strategies had congruences in the number of cases and locations of spatial clusters as well as high co-occurrence with confirmed TAHD at the GMU level, and (iii) discrepancies were found in detections at the GMU level between Confirmed Cases and apparent cases from CS strategies, including within secondary clusters identified by spatial analyses.

Washington's CS strategies provided new and supporting insights into the spatial distribution of TAHD by identifying spatial clusters of apparent disease. Apparent cases were first reported and remained concentrated in GMUs in the south-central portion of the study area. This portion of Washington has high public awareness of TAHD [43], and many of these GMUs were within the original study area, or index area, where the hoof disease was first identified and investigated [22, 25]. This concentration of apparent cases generally aligns with a high number of Confirmed Cases in this region [27], which were collected, in part, to aid those initial investigations. Our use of spatial scan statistics identified local clusters of apparent disease that can be useful targets for management actions by providing inferences beyond simple visualization of data [36–38]. Primary clusters from each CS dataset (and the core-affected area) contained portions of the index area. This result supports previous evidence that TAHD is established in this region. There were, however, some differences in the GMUs comprising primary clusters from each CS dataset. The cluster of Hunter Reports contained more than double the number of GMUs found in the cluster of Public Observations. Reasons for these differences are possibly numerous and warrant further investigation of reporting behaviors by the general public versus hunters. Differences could be associated with, but not limited to, restricted visibility of elk by the general public, hunting access, and reporting fatigue.

Community science strategies also revealed signs consistent with the expansion of TAHD over time. Apparent TAHD cases were broadly recorded across GMUs in the inaugural year that CS surveillance strategies were deployed. After 2012, Public Observations of limping elk appeared to have expanded in spatial distribution over time from the index area. The expansion of apparent cases from this CS strategy may be clearer because of its longer collection period relative to Hunter Reports (2016–present). Still, several secondary clusters from both CS sources were observed directly adjacent to the index area, as would be expected with the spread of a transmissible infectious disease such as TAHD [26]. However, spatial scan statistics applied to each CS strategy also identified a distant cluster in GMUs from the North Cascades elk herd area (Figure S4). The source of TAHD in this disjunct area is unknown. It is currently unclear whether TAHD distribution represents an expansion from the index area through natural or human-assisted movement or multiple sites of origin, e.g., potential spillover events from livestock. The disease may have been unintentionally introduced in 2003 or 2005 when, prior to disease discovery, elk were translocated from the Mount St. Helens herd to the North Cascades to increase herd size [44]. However, apparent geographic leaps in TAHD occurrence were also detected in other areas (e.g., Idaho and California [27]) with no known human-assisted movement of live elk. Restriction on the movement of cervid carcasses from affected areas is commonly employed in attempts to limit the spread of chronic wasting disease-associated prions [45], although the number of states where chronic wasting disease has been detected continues to increase. Similarly, a regulation prohibiting the transport of hooves from carcasses of elk harvested in affected GMUs (WAC 220-413-200) failed to prevent the expansion of TAHD in Washington. Although best practices support the proper disposal of potentially infectious material from carcasses, the causative agent(s) of TAHD may not survive for extended periods outside living hosts [26, 46]. If this is the case, disease expansion is more likely occurring through exposure to or movement of live animals.

The combination of congruences in CS strategies found using a range of analyses and evidence of high co-occurrence with confirmed TAHD cases lends support for the use of CS sources. The number of apparent cases identified by CS strategies were moderately correlated and agreed with each other during the 6-year period when both datasets were available for direct comparison. The two CS datasets also identified spatial clusters in similar GMUs across the study area. Community scientists may misattribute other unrelated causes of limping or hoof abnormalities (e.g., trauma, foreign body, metabolic) to TAHD, but these occurrences are assumed to be relatively constant. Further, observed congruences occurred despite CS strategies collecting data on different visual signs of disease. The combination of this evidence and our observation of high co-occurrence between apparent cases and Confirmed Cases provides confidence in CS strategies appropriately identifying TAHD in our study area. While the CS datasets might not have been completely independent from hoof submissions and subsequent case confirmation (e.g., biologists increasing vigilance to collect samples for case confirmation following public reports), the lack of a systematic sampling strategy for the latter during the study prompted the co-occurrence analyses to examine the association between the datasets at the GMU level. Most conventional validation (or verification) of CS observations requires comparable data collected by scientists [8, 20], but acquiring sufficient “gold standard” data has not been feasible for labor-intensive laboratory confirmations or for opportunistic sightings that often occur on private lands. Instead, observations of abnormal hooves by hunters provided a useful alternative as apparent TAHD cases. As discussed by Wild et al. [30], the accuracy of hunter observations was highly specific (96%) for identifying uninfected hooves and hooves with severe lesions (92%) when compared to visual examination by experts (WDFW, *Unpubl. data*). These comparisons also suggested Hunter Reports underrepresent TAHD prevalence because hunters often missed early lesions. Public Observations may be similarly biased toward advanced disease because limping and lameness in captive research elk increased (and thus became more obvious) with lesion severity [26].

We show that collecting observations of limping elk may be a useful early indicator for the presence of TAHD, as indicated by such apparent cases predating TAHD confirmation in GMUs by several years. The online reporting tool facilitated data collection at relatively high spatial and temporal resolutions. These details can aid in focusing sample collection for diagnostic examinations and confirming TAHD. In some other states without public reporting systems aimed at detecting TAHD, the disease was first identified through Confirmed Cases submitted for enhanced regional surveillance [27]. Managers in at-risk locations for TAHD occurrence may consider implementing education to increase disease awareness and CS surveillance to enhance early detection.

The opportunity to detect temporal trends in apparent case occurrence, both seasonally and across years, is another advantage of this continuously accessible CS strategy. We found an apparent seasonality in observations of limping elk, but the reason for observed increases in Public Observations during harvest seasons requires further investigation. Given the opportunistic nature of the dataset, our analyses were restricted to describing patterns in reporting rather than patterns of disease. Seasonality in reporting could be indicative of time periods with higher risk of TAHD, times with higher movement and visibility of elk (e.g., during the rut), or seasonal activity of observers. More participant information, such as affiliations with recreational activities and reporting behaviors, could provide confidence in differentiating disease trends from patterns in observations of limping elk [47, 48]. For instance, whether disease losing novelty in an area reduces public motivation to report observations (i.e., reporting fatigue) or determining how many observers of limping elk were also hunters could help disentangle temporal trends in limping elk from possible reporting biases [47, 49], such as attributing observations to hunter activity. Hunters generally spend more time outdoors before and during fall harvest seasons to scout areas and hunt. These outings could lead to opportunities for observing limping elk and explain the seasonality we observed if hunters are routinely using the online reporting tool. Additionally, the public's threshold and motivations for reporting limping elk are unknown but could be useful for interpreting evidence of TAHD spread in the study area. That is, changes in either observer awareness of TAHD or abilities and motivations to report limping elk could also result in the expanding distribution of apparent cases [50]. Understanding such motivations and reporting behaviors will be important for keeping CS participants engaged in the data collection process [51, 52] as well as achieving deeper inferences from their data.

Data from Hunter Reports, as currently collected, are valuable for monitoring TAHD prevalence and understanding trends in disease. Female elk had relatively higher odds for Hunter-Reported hoof abnormalities than males, suggesting a sex-specific predilection in TAHD occurrence. This higher relative prevalence in females may be a result of increased susceptibility to the disease, risk of transmission from social grouping behaviors, or differences in habitat selection [5, 53]. However, we cannot exclude potential sampling bias from differential harvest opportunities due to hunting regulations. Additionally, Hunter Reports allowed us to identify trends in prevalence for individual elk herd areas in the 2016–2021 harvest seasons. These trends provide a useful baseline of prevalence trajectories for comparisons and evaluations of future management actions. Although minor changes in hunting regulations occurred over time, we observed increasing trends in apparent TAHD prevalence for Mount St. Helens and North Rainier elk herd areas and a decreasing trend in prevalence for the Willapa Hills herd area. Prevalence estimates for other herd areas over time were less clear, presumably due to variability from small sample sizes given low hunting effort/success.

Enhancing the data collected from Hunter Reports in three ways could allow additional inferences for management. First, some Washington hunters do not submit Hunter Reports after the harvest season. The number of hunters who fail to report is estimated to be substantial. Likewise, of the successful hunters who do submit a report, most (about 92%) do not provide fine-scale spatial coordinates of their harvest locations as requested in follow-up surveys (WDFW, *Unpubl. data*). This results in most Hunter Reports being collected at the coarse spatial resolution of GMUs, which can hinder examinations of fine-scale spatial patterns (e.g., hotspots and spread patterns [36]). Limited fine-scale data restricts investigations of influential risk factors for disease occurrence [54–57] and predicting risk on landscapes [58, 59]. Collaboration with trusted organizations may be a way to promote the collection of these missing data [43, 60]. Communicating the importance of hunter information for TAHD surveillance and decision-making and providing practical examples of how such data are used may be helpful in promoting collaboration and augmenting reporting [61, 62]. Also, framing the importance of hunter data as a means of empowering conservation and promoting long-term hunting opportunities should be continued and expanded [60]. Second, more detailed metadata on elk age could allow adjustment for a possible confounding variable in sex-specific prevalence. If the probability of acquiring TAHD increases with age, as observed in some other infectious wildlife diseases [63], differences in age-related harvest (or vulnerability to harvest) may have had a confounding effect on observed sex-related differences in apparent prevalence. That is, considering males rarely reach older ages due to harvest pressures in western Washington, higher prevalence in females may relate to their age rather than sex. Finally, we examined prevalence at the relatively coarse scale of Washington's elk herd areas. These designations are administrative areas designed for coordinated elk management rather than true delineations of elk populations; therefore, though logistically challenging to obtain, estimates of elk herd abundance would provide more realism to trends in prevalence at the herd level [64].

We found that CS data provide complementary information to the regional gold standard surveillance strategy of confirming cases through histologic diagnosis of submitted hooves. The absence of Confirmed Cases in some GMUs with Public Observations and Hunter Reports likely resulted from a lack of sample submission for diagnostic confirmation rather than a “false positive” area designation by CS strategies in our study area. In contrast to areas with TAHD-negative submissions, several GMUs within the southern portion of the study area lacked Confirmed Cases despite biologists and stakeholders suspecting the presence of TAHD given observations of elk with characteristic signs of TAHD and proximity of GMUs to areas with confirmed disease. Confirming TAHD may be perceived as having little practical benefit for managing resources in an area if managers are aware of its likely occurrence, particularly when coordination and resources are needed to submit hoof samples for laboratory confirmation of TAHD cases [27]. However, disease confirmation may have the added value of positive social and biological impacts. Stakeholders' trust in agency vigilance in disease management may be enhanced when concurrence is observed between areas of Confirmed Cases illustrated on disease distribution maps and areas where they suspect TAHD occurs based on observations of elk with signs of TAHD. Also, analyses of disease spread or origins (e.g., [65]) may be improved by documenting geographic distribution in disease occurrence in a more systematic manner. Finally, as evidenced in this study, the co-occurrence of confirmed TAHD with apparent cases can provide a means of supporting CS strategies. In addition to providing initial confirmation of TAHD in new areas, continued monitoring through case confirmation in known areas provides evidence-based information to corroborate CS surveillance strategies and limit chances for false positives from other diseases with similar signs that might occur over time.

## 5. Conclusion

Given the obvious visual signs of disease in a species that is highly valued for wildlife viewing and hunting, TAHD presents an ideal system for conducting disease surveillance with the assistance of community scientists. We report the expanding spatial distribution of apparent cases from the core-affected area in southwestern Washington to additional GMUs. Our analyses provide insights for developing wide-reaching and complementary TAHD surveillance programs. Considering Public Observations of limping elk generally predated confirmations of TAHD by several years, wildlife agencies may benefit from raising public awareness of the disease and providing an online platform for collecting observations of limping elk as an early warning indicator of TAHD, as has been done by WDFW. Such reports from the public may trigger the collection of samples for diagnostic examinations, which are required to confirm TAHD in an area and corroborate observations from CS strategies. Our results show that TAHD prevalence and trends of disease can be broadly understood with Hunter Reports of hoof abnormalities. However, deeper understandings of TAHD dynamics and spread could be supported by ensuring Hunter Reports collect enhanced information with fine spatial resolution and, when possible, detailed metadata of elk demographics. Consistent with other CS programs, efforts to understand the motivations and identities of participants and to communicate the need for data will be needed for long-term TAHD surveillance and monitoring in Washington and likely other areas with TAHD.

## Figures and Tables

**Table 1:** Data properties for three Washington treponeme-associated hoof disease surveillance strategies, 2009–2022.

	Public Observations	Hunter Reports	Confirmed Cases
Description	Public observations of limping elk reported on online mapping tool maintained by WDFW	“Yes”/“No” responses to whether hunters observed hoof abnormalities on harvested elk	Expert-solicited elk hoof samples used in formal diagnostic investigations
Data type, collection scheme	Presence-only, passive	Presence–absence, active	Presence–absence, mixed active/passive
Spatial scale	Coordinates	Game management unit (GMU)	GMU/county/coordinates
Temporal scale, collection period	Continuous, 2012–present	Annual (seasonal), 2016–present	Continuous, 2009–present
Diagnostic sensitivity	Unknown	60% overall^1^	79%^2^
Diagnostic specificity	Unknown	96%^1^	100%
Geographic extent	Washington	Washington	Regional (Multi-state)^3^

*Note*. ^1^Unpublished data, Washington Department of Fish and Wildlife (WDFW). Sensitivity of Hunter Reports varied by lesion severity and is with respect to gross observation by an expert observer; Wild, Sargeant et al. [30]. ^2^Sensitivity from a similar technique in bovine digital dermatitis model by Krull et al. [31]. ^3^i.e., Washington, Idaho, California, and Oregon; Wild, Taylor et al. [27].

## Data Availability

The datasets described in this article are not immediately accessible as they are subject to restrictions and not publicly available. However, they were obtained for the current study under a data sharing agreement. The data can be obtained directly from the authors upon reasonable request and with permission from Washington Department of Fish and Wildlife (kyle.garrison@dfw.wa.gov).

## Supplementary Materials

Supplementary Materials Figure S1 online reporting tool developed and maintained by the Washington Department of Fish and Wildlife (WDFW). Figure S2: Washington's treponeme-associated hoof disease (TAHD) surveillance data and herd areas. Figure S3: spatial correlograms for Hunter Report and Public Observation data. Figure S4: secondary clusters detected in CS strategies from spatial scan statistics. Table S1: binomial generalized linear model outputs for trends in herd-level prevalence over time (years).
