# Supplementary material for: Community Science Strategies Reveal Distributional Patterns of Treponeme-Associated Hoof Disease in Washington Elk (Cervus canadensis)
Source: Transbound Emerg Dis. 2023 Dec 14;2023:6685108. doi: 10.1155/2023/6685108 (PMC12017226; doi:10.1155/2023/6685108)
Supplement: Supplementary Materials — Figure S1 online reporting tool developed and maintained by the Washington Department of Fish and Wildlife (WDFW). Figure S2: Washington's treponeme-associated hoof disease (TAHD) surveillance data and herd areas. Figure S3: spatial correlograms for Hunter Report and Public Observation data. Figure S4: secondary clusters detected in CS strategies from spatial scan statistics. Table S1: binomial generalized linear model outputs for trends in herd-level prevalence over time (years). [file 6685108.f1.docx]

Supplementary Materials for “Community science strategies reveal distributional patterns of treponeme-associated hoof disease in Washington elk (*Cervus canadensis*)” by Winter et al.


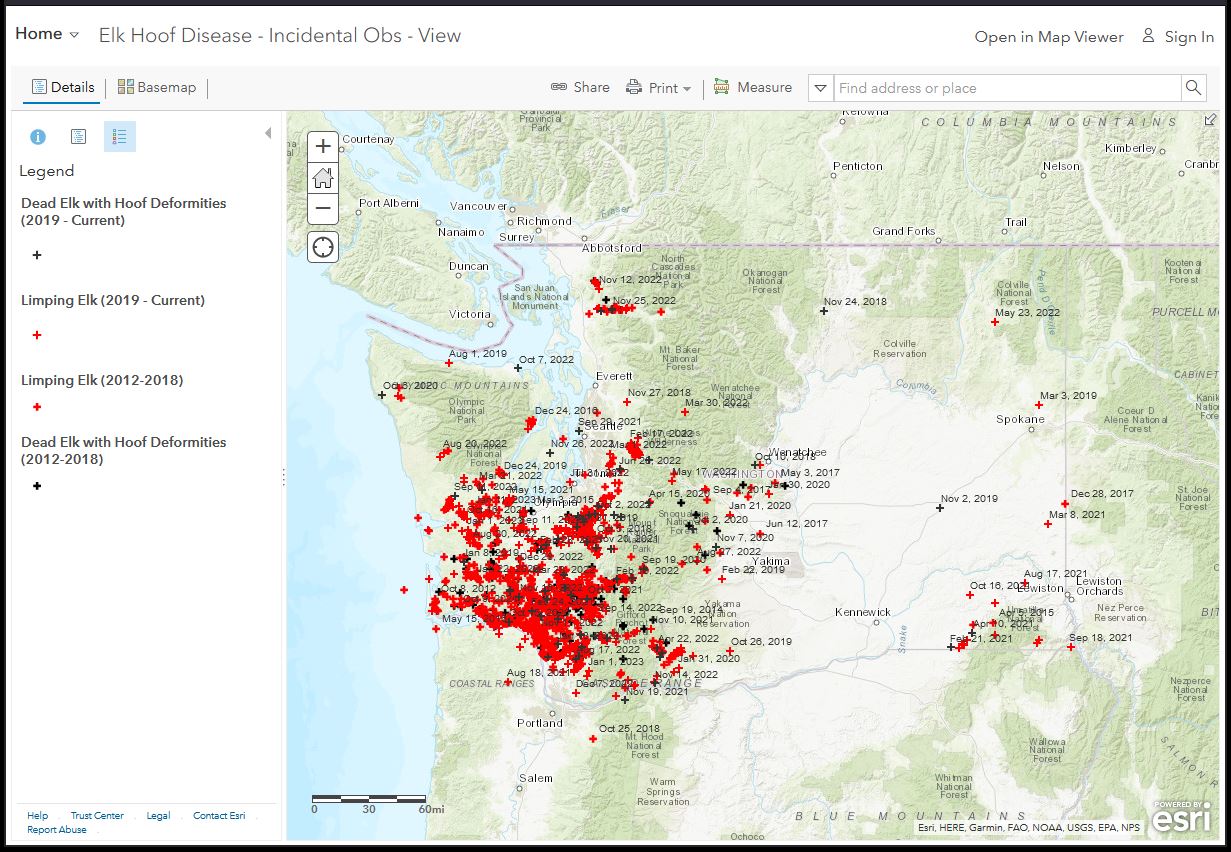


**Figure S1:** **Online reporting tool developed and maintained by Washington Department of Fish and Wildlife (WDFW).** Image shows dashboard of publicly visible data collected with the Public Observations CS strategy with limping elk records shown in red and dead elk with hoof deformities in black plus signs.


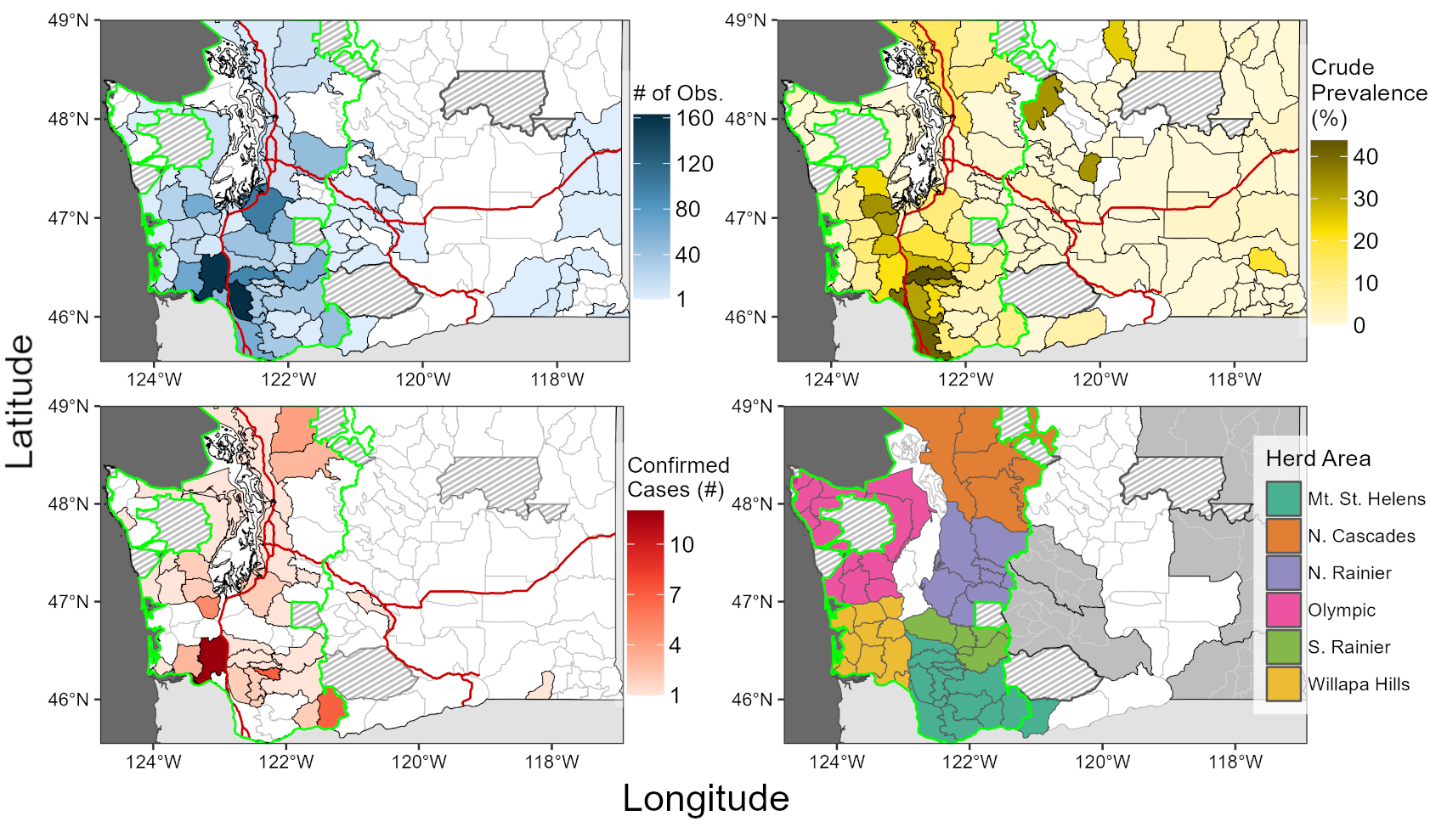


**Figure S2: Washington’s treponeme-associated hoof disease (TAHD) surveillance data and herd areas.** Panels show western concentration in the distributions of public observations of limping elk (blues; top left), hunter reported prevalence (yellows; top right), number of confirmed TAHD cases (reds; bottom left), and elk herd areas (lower right). The study area (outlined in fluorescent green) was selected due to higher concentrations of apparent cases based on public observations and hunter reports (community science data), and included the area of primary investigation by WDFW (index area) indicated by those GMUs with higher numbers of confirmed cases. The latter coincides with the GMUs containing western Washington’s elk herd areas (colored polygons in green westside boundary; bottom right). The crude prevalence estimates are not adjusted by sample sizes and thus do not reflect uncertainty in estimates. Similarly, the number of confirmed cases or public observations are not based on sampling effort. We include interstate highways (red lines) for reference. Areas outside Washington Department of Fish and Wildlife jurisdiction are shown in striped polygons and neighboring states (Oregon to south, Idaho to east) in gray.


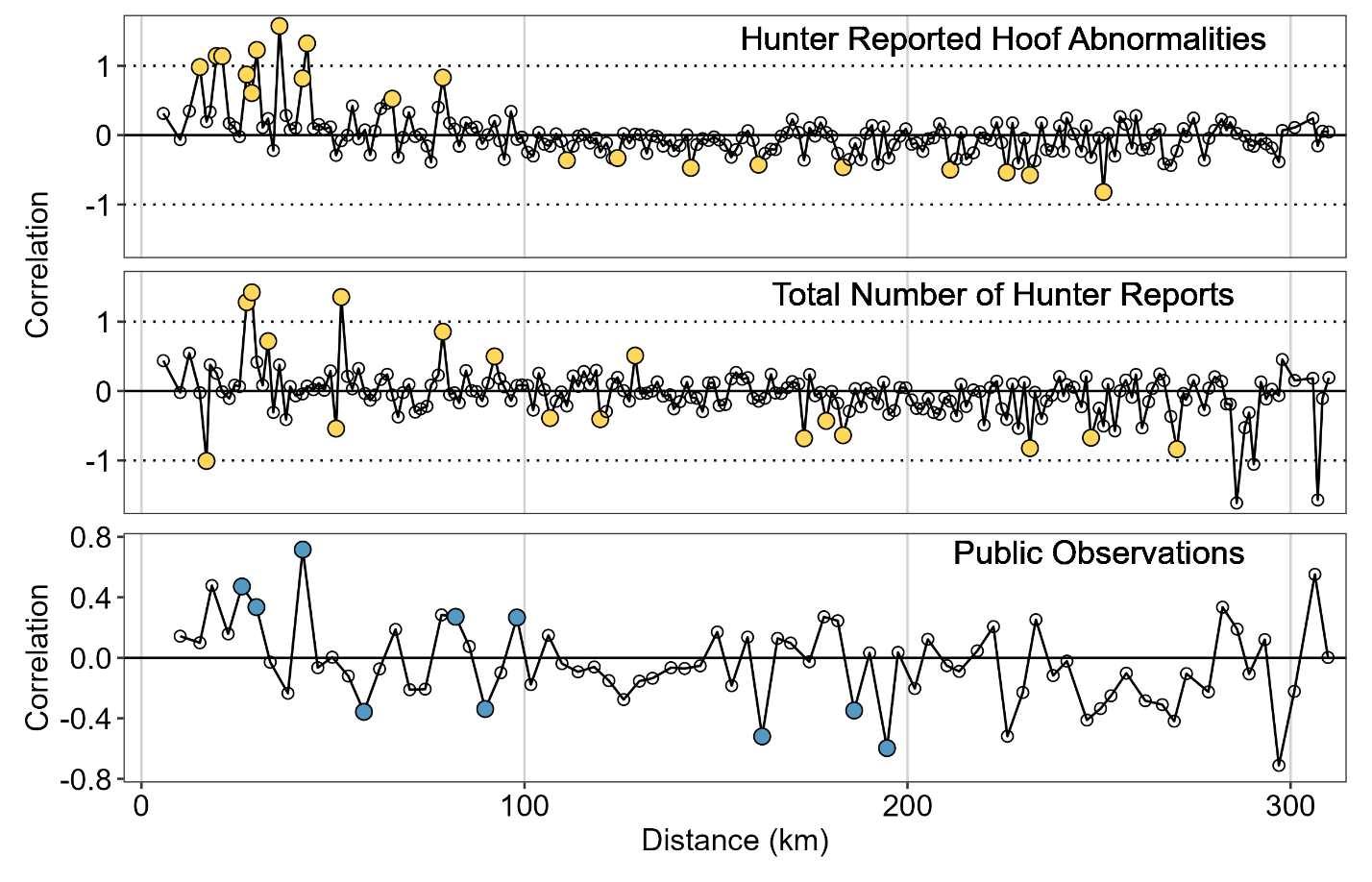


**Figure S3: Spatial correlograms for Hunter Report and Public Observation data.** Panels show spatial correlograms with lagged distance in kilometers (x axis) and Moran’s I correlation coefficients (y axis). Statistically significant correlations are denoted with filled circles. Positive spatial correlations were consistently found at shorter lagged distances for hunter reported hoof abnormalities (top). We found additional significant spatial autocorrelation in hunting success in general (middle) and Public Observations (bottom).


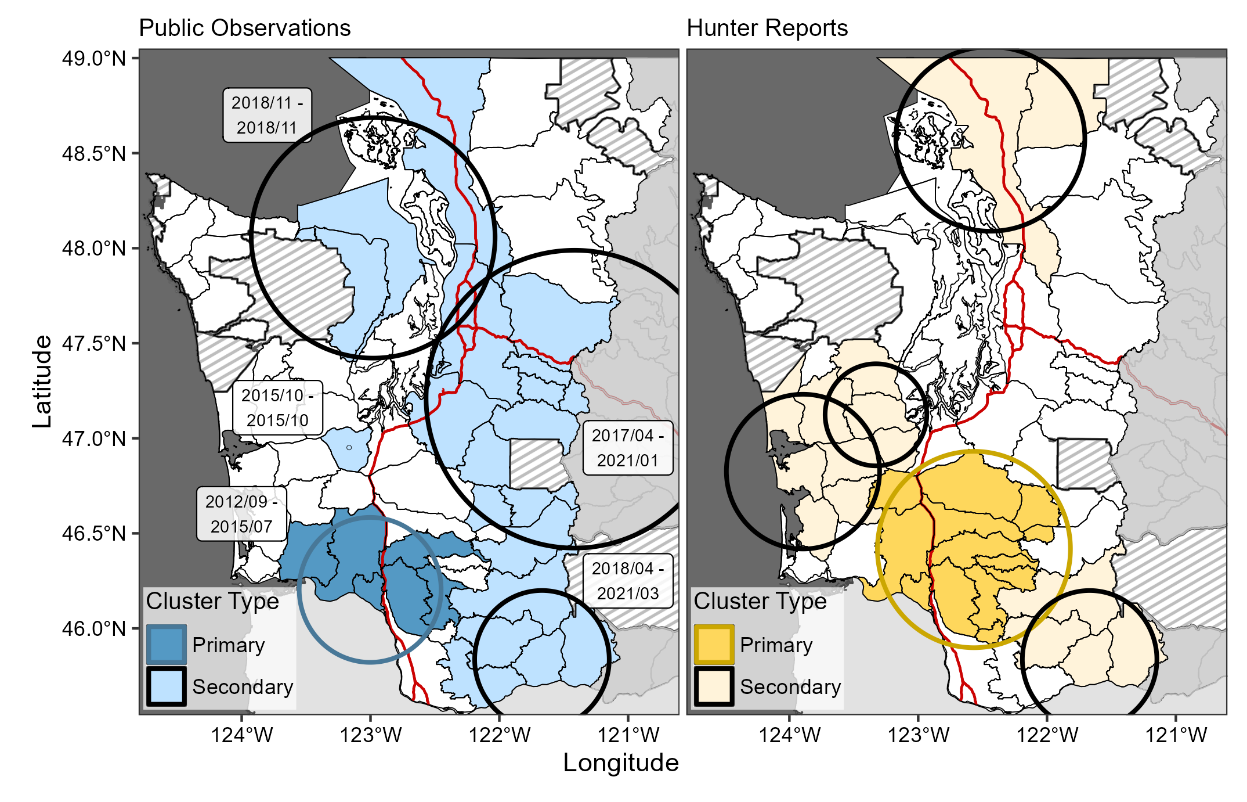


**Figure S4: Secondary clusters detected in CS strategies from spatial scan statistics. Left:** Dark blue polygons represent game management units (GMUs) that contained the primary cluster of Public Observations (blue circle) while lighter blue GMUs and black circles represent significant secondary space-time clusters. The durations of clusters (year/month shown in white rectangles) are largely consistent with an expanding distribution of reports over time. **Right:** Yellow polygons represent the GMUs contained within the most likely Hunter Reports spatial cluster (golden circle) and GMUs containing secondary clusters are shown in lighter yellow polygons and black circles. Secondary clusters in Hunter Reports were noted in similar GMUs to Public Observations seen in the left panel. For landmarks, red lines represent interstate highways and striped polygons represent jurisdictions outside of Washington Department of Fish and Wildlife GMUs, including tribal reservations and national parks.

**Table S1: Binomial generalized linear model outputs for trends in herd-level prevalence over time (years).** The reference category for the herd variable is the Mount St. Helen's herd.

|  | Estimate | Standard Error | Z value | P-value |
| --- | --- | --- | --- | --- |
| (Intercept) | -95.241 | 44.219 | -2.154 | 0.031 |
| HerdNCascades | 223.385 | 263.977 | 0.846 | 0.397 |
| HerdNRainier | -261.573 | 111.149 | -2.353 | 0.019 |
| HerdOlympic | 18.258 | 164.099 | 0.111 | 0.911 |
| HerdSRainier | -133.657 | 106.207 | -1.258 | 0.208 |
| HerdWillapa | 218.904 | 70.606 | 3.1 | 0.002 |
| Year | 0.047 | 0.022 | 2.124 | 0.034 |
| HerdNCascades:Year | -0.111 | 0.131 | -0.849 | 0.396 |
| HerdNRainier:Year | 0.129 | 0.055 | 2.342 | 0.019 |
| HerdOlympic:Year | -0.01 | 0.081 | -0.121 | 0.904 |
| HerdSRainier:Year | 0.066 | 0.053 | 1.254 | 0.21 |
| HerdWillapa:Year | -0.109 | 0.035 | -3.113 | 0.002 |
